# Safety and family satisfaction of a home-delivered chemotherapy program for children with cancer

**DOI:** 10.1186/s13052-021-00993-x

**Published:** 2021-02-26

**Authors:** Lucia De Zen, Irene Del Rizzo, Luca Ronfani, Francesca Barbieri, Marco Rabusin, Roberto Dall’Amico, Egidio Barbi, Margherita Robazza

**Affiliations:** 1 Pediatric Palliative Care and Pain Service, Institute for Maternal and Child Health Burlo Garofolo, Trieste, Italy; 2 Clinical Department of Medical, Surgical and Health Sciences, University of Trieste, Trieste, Italy; 3 Department of Clinical Epidemiology, Institute for Maternal and Child Health Burlo Garofolo, Trieste, Italy; 4 Pediatric Department, Azienda Sanitaria Friuli Occidentale, Pordenone, Italy; 5 Pediatric Department, Oncology and Hematology Unit, Institute for Maternal and Child Health Burlo Garofolo, Trieste, Italy; 6 Pediatric Department, Institute for Maternal and Child Health Burlo Garofolo, Trieste, Italy

**Keywords:** Chemotherapy, Home assistance, Quality of life, Childhood cancer

## Abstract

**Background:**

Home chemotherapy programs for children with cancer are safe and feasible, and their impact on the quality of life has been reported in different countries. A home chemotherapy program was implemented between 2011 and 2019 in an Italian region. This pilot study investigates its safety and feasibility, along with parental satisfaction.

**Methods:**

Patients between 0 and 18 years diagnosed with malignancy were included. Deceased patients and patients whose families moved abroad or interrupted contact with the service were excluded. Adverse events comprised immediate deterioration of the patient’s condition, equipment failure, errors in drug storage, dose or patient identification and personnel safety issues. Parental satisfaction was explored through an email survey of 32 Likert-type and short open questions.

**Results:**

Thirty-five patients received 419 doses of intravenous chemotherapy at home (cytarabine, vincristine, vinblastine). No adverse events were reported. Twenty-three families out of 25 eligible completed the survey. Most reported being “very satisfied” with the possibility of maintaining a work/domestic routine and reducing time and financial burden of hospital access. Most were “very satisfied” with the opportunity for their child of being less troubled by the treatment. Besides, most reported being “very satisfied” with the chance for healthy siblings of maintaining their routine and coping with their brother/sister’s disease. Most perceived the program as safe. All families recommended extending the program to all children in the region.

**Conclusions:**

This first Italian study supports home chemotherapy as safe and effective, positively influencing the quality of life for children and their families.

**Supplementary Information:**

The online version contains supplementary material available at 10.1186/s13052-021-00993-x.

## Background

While cancer is still a leading cause of death in childhood and adolescence in high-income countries [1, 2], advances in the survival rates arise interest in the quality of life of children and their families during the treatment [3]. Home care programs for children with cancer have been proved effective in enhancing the quality of life while providing safe treatments and reducing hospitalization-related costs [4]. More specifically, the delivery of intravenous chemotherapy at home is described as safe and feasible for both adults and children, with the first results dating back to the Nineties [5–7]. Home chemotherapy requires adherence to strict conditions, related to the treatment protocol and phase, home environment and location, caregiver motivation and training, and patient characteristics [8]. Moreover, these programs are cost-effective and favorably impact on the quality of life of patients and families [9].

In Italy, a successful experience of home care for children with hematological malignancies and after hematopoietic stem cell transplant at the “Giannina Gaslini” Institute in Genoa has been published: children were taken care at home with blood drawing, central venous catheter (CVC) medications, supportive treatment and end-of-life care, but the program did not include chemotherapy administration [10, 11].

In the Friuli Venezia Giulia Region, since 2011, a home assistance program run by the Pediatric Home Care Team (PHCT) from the Pediatric Department at the Spoke Hospital of Pordenone takes care of children with complex needs, both oncological and not, from the Pordenone province and the nearby Udine and Venice provinces. The team provides different services at home, including intravenous chemotherapy, blood tests, CVC medication, assessment of compliance with oral medications and dose adjustments on behalf of the Hub Oncology Centre, supportive treatment (including antibiotic and antifungal therapy, transfusions, pain management), psychological support for children and families, and end-of-life care. Home chemotherapy delivery has been one of the main activities since it started in 2011. Then, the project gradually expanded, comprising children with non-oncological complex needs. A pediatrician and two pediatric nurses, all from the Pordenone hospital Pediatric staff, and a psychologist work 5 days a week from 8.00 am to 4.00 pm, with the pediatrician available on call until 8.00 pm. For emergencies, at night and during the weekends, patients rely on the on-duty staff at the Pediatric Department of the Pordenone Hospital. The Pordenone hospital thus works as a Spoke hospital, admitting children for the complications of oncological treatments (febrile neutropenia, mucositis), but also planning the activity of the PHCT. The drugs chosen for home administration are cytarabine, vincristine and vinblastine because they have sufficient stability, do not require prolonged infusions or any specific support therapy (i.e. hyper-hydration) and do not carry a significant risk of immediate adverse reactions (i.e. anaphylaxis or acute toxicity) [12, 13]. The Pharmacy Department of the Oncology Centre in Aviano prepares the drugs under sterile conditions and guarantees safe transport and the Medical Board of the Hospital approved a written protocol for their storage, transportation, delivery and disposal. The mean time spent on each home visit is 45 min, including physical examination, drug administration (slow push technique over 5 to 10 min), counselling, and other procedures, i.e. CVC care or blood sample drawing. As a result, the actual time spent on the chemotherapy administration is less than 15 min of the whole visit.

This research aims to explore the safety and the impact of home-delivered chemotherapy on the quality of life of children and their families.

## Methods

We performed a retrospective evaluation on children who received chemotherapy at home by the PHCT in the Local Health Authority of Pordenone (*Azienda Sanitaria Friuli Occidentale*, ASFO) in Friuli Venezia Giulia, Italy.

Inclusion criteria were: age between 0 and 18 years, diagnosis of malignancy, intravenous chemotherapy carried out at home by the Pediatric Department of ASFO on behalf of the Hub Pediatric Oncology and Hematology Centre between 01/06/2011 and 31/05/2019. We excluded deceased patients and patients whose families moved abroad or interrupted their contact with the Pediatric Department.

The primary outcome was the frequency of adverse events; the secondary outcome was family satisfaction measured through questionnaires.

For this research, we focused only on intravenous chemotherapy delivered at home, avoiding questions on oral chemotherapy, diagnostic procedures, support therapy (i.e. transfusions, antibiotic, pain treatment) or end-of-life care.

We retrospectively collected informations on family socio-demographics (including nationality and language, primary caregiver’s education and profession, spouse’s education and profession), patients’ age at diagnosis, number of family members, distance from the nearest hospital and the Hub Oncology Centre, number of working days lost due to therapies, the possibility of having paid leave for the primary caregiver and obtaining financial support from charities in order to depict a broader picture of the families.

Regarding the primary outcome, we reviewed the clinical records to document adverse events. Adverse events include immediate acute deterioration of patient’s condition (i.e. anaphylactic reactions, drug extravasation or any other critical condition requiring emergency transport to the hospital), equipment failure (i.e. CVC malfunctioning), errors in drug transport or storage, staff safety issues (i.e. accidental contamination with cytotoxic drugs or needle stick), errors in dosing or patient identification. We did not focus on predictable side effects of chemotherapy (i.e. nausea and vomiting, fever, neutropenia) because we assumed that they occurred at the same rate as the treatment administered in the hospital setting.

As for family satisfaction, we collected qualitative data on the impact of home-delivered chemotherapy on the family routine and the overall quality of life, and the perception of safety and effectiveness. We used a 5-item Likert-type scale ranging from “not at all satisfied/completely disagree” to “very satisfied/strongly agree” to assess parental satisfaction on different aspects and short open questions. The full questionnaire is provided in the Supplementary Information files (Supplementary file 1).

We sent an invitation email to eligible families with the project description and a link to the survey. The Hospital Revisory Board approved the questionnaire. Parents filled a consent form for clinical charts revision for scientific purposes when they were first admitted to the hospital. The email with the link contained a specific consent question for anonymized data collection.

We performed only a descriptive analysis. Continuous data were presented as median and interquartile range (IQR); categorical data (including the Liker-type questions) as number and percentage.

## Results

**Fig. 1:**
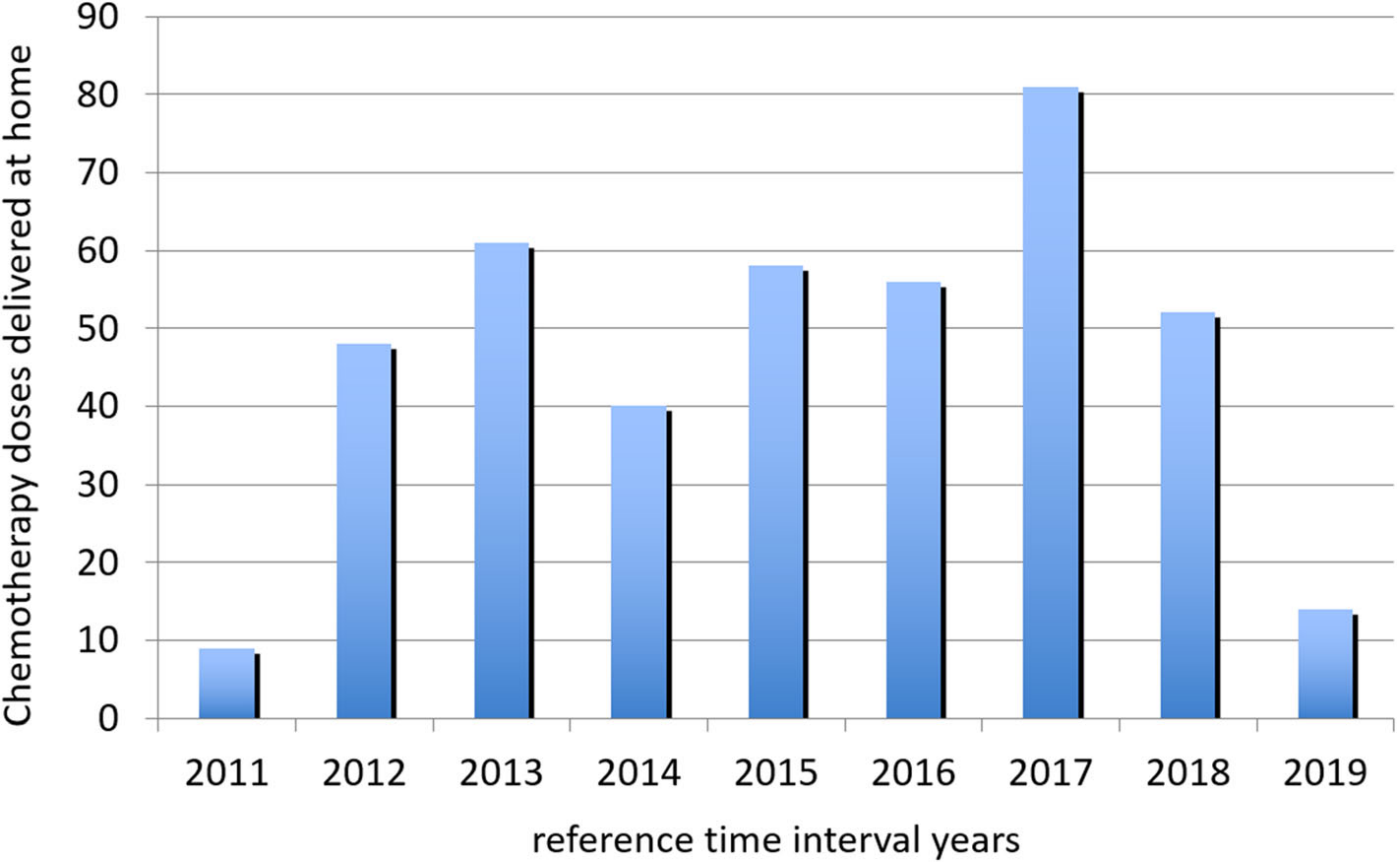
Distribution of the chemotherapy doses delivered at home for each year of the reference time interval

We collected data on primary and secondary outcomes between February, 1st 2020 and April, 30th 2020. As for the safety analysis, 35 patients met the inclusion criteria, 22 males and 13 females, with a median age at diagnosis of 6 years (range 1–15 years, IQR 5 years). The most common diagnosis was acute lymphoblastic leukemia (ALL) with 32 cases, followed by Wilms tumor (2 cases) and soft tissue sarcoma (one case). Five patients with ALL experienced disease relapse. We, therefore, considered 40 chemotherapy protocols for a total amount of 419 doses administered at home in the reference time interval. Figure 1 summarizes the distribution of the chemotherapy doses delivered at home for each year of the reference time interval.

All patients had a central venous catheter inserted at the Hub Oncology Centre.

We did not report any acute deterioration of the patients’ condition, nor equipment failure, nor errors in drug transport, storage or disposal, nor errors in dosing, patient identification or staff safety issues.

As for the secondary outcomes (satisfaction), we first considered all 35 patients. We excluded three patient for contact problems, three patients who completed their treatment abroad and four deceased patients. In the end, we enrolled 25 families to participate in the questionnaire. The diagnoses were ALL (22 cases), Wilms tumor (two cases) and soft tissue sarcoma (one case). There were 16 males and nine females.

Twenty-three families answered the anonymous questionnaires (dropout rate 8%). Median age at diagnosis of children for the response group was 5 years (range 1–15 years, IQR 4). Most children were less than 7 years old at the time of diagnosis.

There were 19 Italian and four foreign families, but all use the Italian language to communicate with the team.

The area covered by the PHCT was 900 km^2^ (Supplementary information file 2). Most families lived within 20 km from the nearest hospital (12/23, 52.2%; median distance from the nearest hospital 12 km, IQR 15 km) and more than 100 km far from the Hub Oncology Centre (13/23, 56,5%, median distance from the Hub Oncology Centre 110 km, IQR 20 km). All families reached the hospital by car. Most families reported a mean hospital access rate of less than five times a month (13/23, 56,5%). Most families (21/23, 91,3%) received financial support from charities or foundations during the therapy. Most caregivers (12/23, 52,2%) reported between 30 and 365 personal days taken from work and most reported to be allowed some paid leave (14/23, 60,9%); a minority of caregivers gave up their job (2/23, 8,7%). The mother was the most frequent primary caregiver (22/23, 91,3%) and also the person who spent the most time with the child during the hospital stay.

**Fig. 2:**
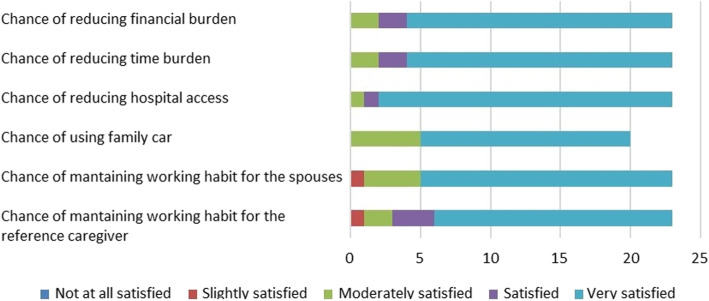
Parental satisfaction related to work, travel and financial issues

**Fig. 3:**
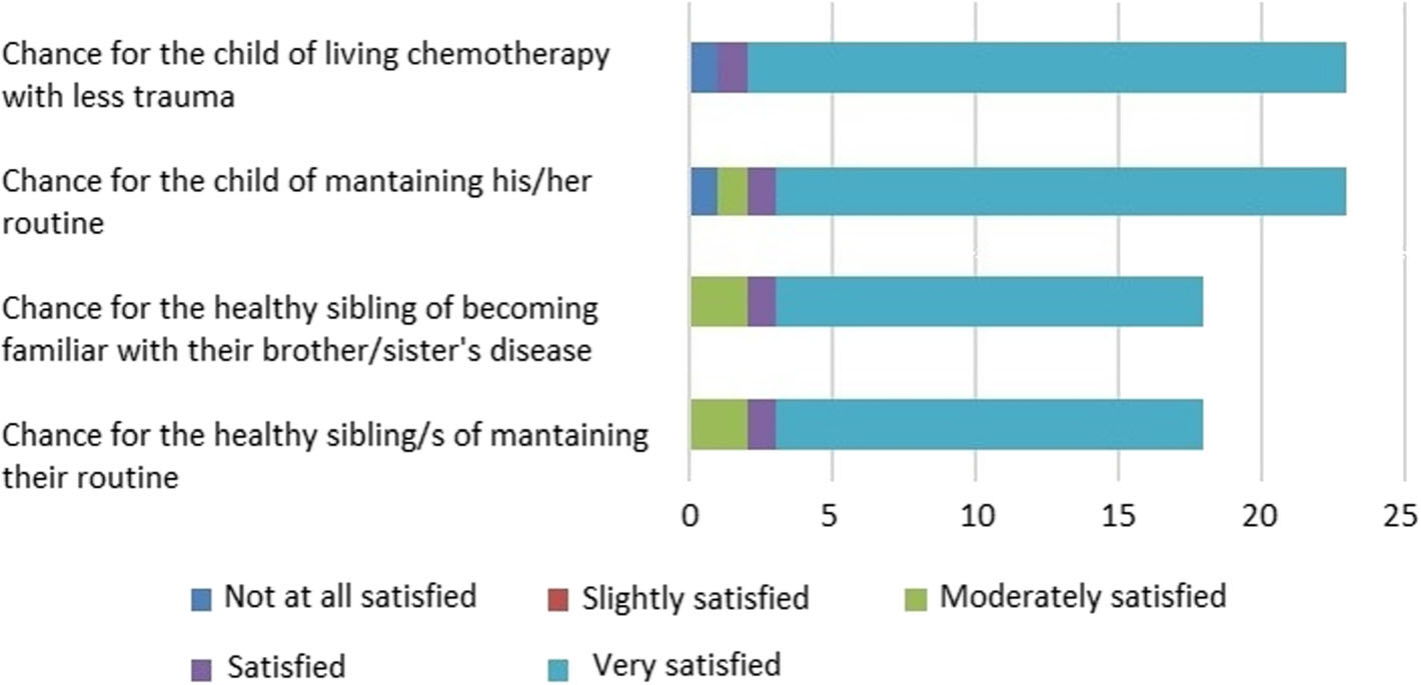
Parental satisfaction related to the psychological impact of the program on the ill child and his/her healthy sibling/siblings

**Fig. 4:**
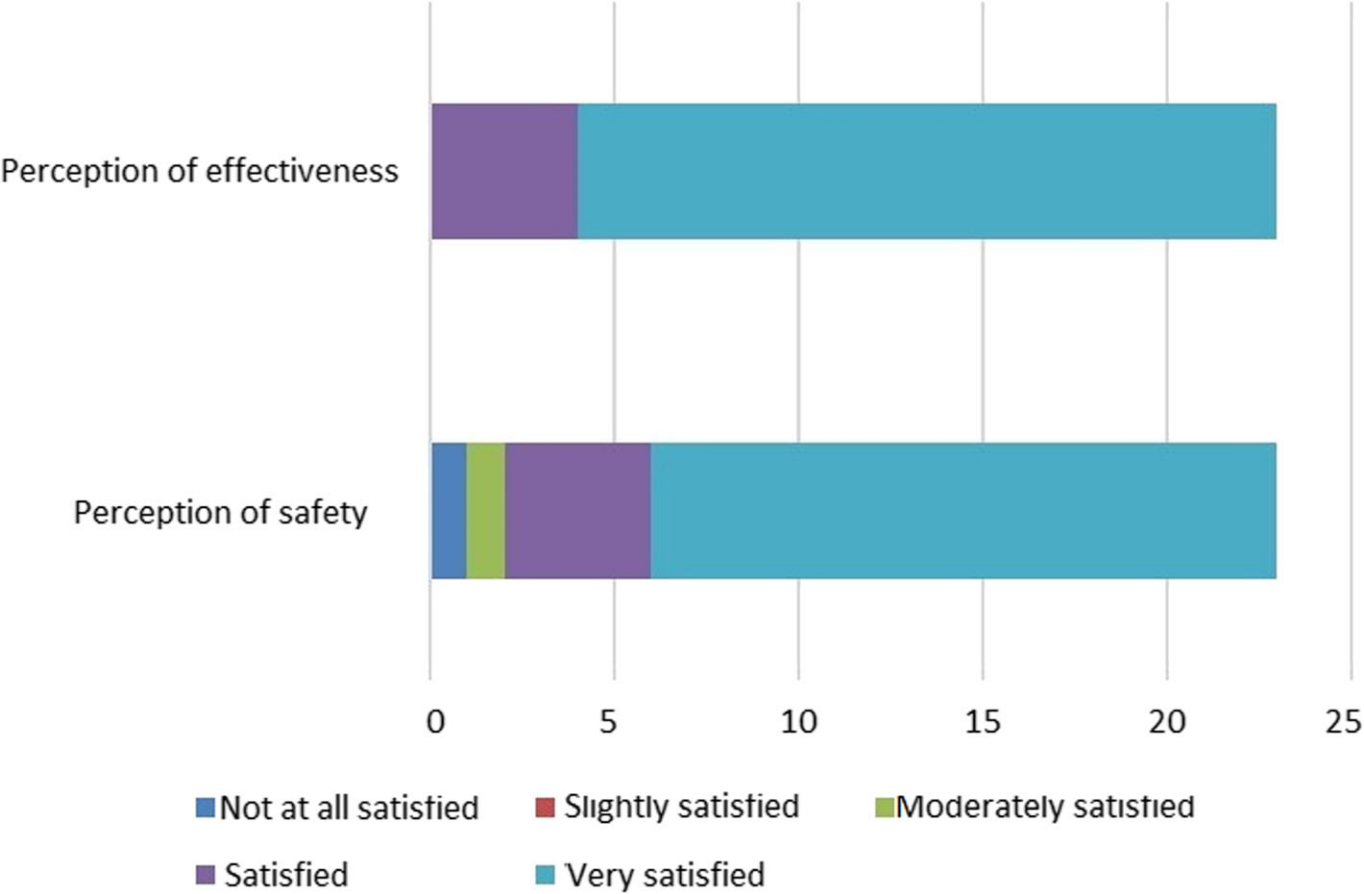
Parental satisfaction related to the perception of safety and effectiveness of the program

Tables 1 and 2 summarize participants’ socio-demographics, travel, and job-related issues. Figures 2 summarizes parental satisfaction related to travel, job and financial issues. In Fig. 3, parental satisfaction associated with the psychological impact on the child and healthy siblings is summarized. Figure 4 summarizes parental satisfaction related to the perception of safety and effectiveness and suggestion on program expansion.

**Table 1:** Participants’ socio-demographics

	N	*%*
Main caregiver (*N* = 23)		
Mother	21	91,3%
Father	2	8,7%
Family nationality (*N* = 23)		
Italian	19	82,6%
Other	4	17,4%
Language adopted with the Service team (*N* = 23)		
Italian	23	100%
Other	0	0%
Main caregiver’s profession (*N* = 23)		
Housewife	5	21,7%
Workman	4	17,4%
Office worker	8	34,8%
Executive manager	2	8,7%
Self-employed	3	13,0%
Entrepreneur	1	4,3%
Spouse’s profession (*N* = 23)		
Housewife	0	0,0%
Workman	6	26,1%
Office worker	9	39,1%
Executive manager	4	17,4%
Self-employed	2	8,7%
Entrepreneur	2	8,7%
Main caregiver’s instruction level (*N* = 23)		
Primary school diploma	1	4,3%
Middle school diploma	2	8,7%
High school diploma	9	39,1%
University degree	9	39,1%
Professional school license	2	8,7%
Spouse’s instruction level (*N* = 23)		
Primary school diploma	1	4,3%
Middle school diploma	3	13,0%
High school diploma	9	39,1%
University degree	2	8,7%
Professional school license	8	34,8%
Total number of family members (*N* = 23)		
3	8	34,8%
4	8	34,8%
5	6	26,1%
6	1	4,3%
Number of other children (*N* = 23)		
None	6	26,1%
1	9	39,1%
2	7	30,4%
3	1	4,3%
Age of the child at the diagnosis (*N* = 23)		
0–3 years	7	30,4%
4–7 years	11	47,8%
8–12 years	2	8,7%
> 12 years	3	13,0%

**Table 2:** Means of transport, distance and parental job-related issues

	N	%
Means of transport used during therapy (*N* = 23)		
On foot	0	0%
Car	23	100%
Taxi	0	0%
Train/bus	0	0%
Average monthly frequency of travels to hospital for visits, procedures and therapies (*N* = 23)		
Less than 5 times a month	13	56,5%
Between 5 and 10 times a month	5	21,7%
More than 10 times a month	5	21,7%
Distance from the nearest hospital (km) (*N* = 23)		
Less than 5 km	7	30,4%
Between 5 and 20 km	12	52,2%
More than 20 km	4	17,4%
Distance from the Hub Oncology Centre (km) (*N* = 23)		
Less than 50 km	2	8,7%
Between 50 and 100 km	8	34,8%
More than 100 km	13	56,5%
Average monthly frequency of absences from work for the main caregiver due to visits, procedures and therapies (*N* = 23)		
< 30 days	5	21,7%
30–365 days	12	52,2%
> 365 days	4	17,4%
Layoff	2	8,7%
Main caregiver allowed some paid leave from work (*N* = 23)		
Yes	14	60,9%
No	9	39,1%
Financial support by charities/foundations (*N* = 23)		
Yes	21	91,3%
No	2	8,7%

Table 3 summarizes open comments and family opinions on the expansion of the program for all children with cancer in the Region.

**Table 3:** Short open comments and agreement with program expansion

**Agreement with program implementation in the Region (*N*** **= 23)**	***N***	**%**
Strongly agree	21	91,3%
Agree	1	4,3%
Moderately agree	1	4,3%
Disagree	0	0%
Strongly disagree	0	0%
**Short open comments (*N*** **= 23)**	***N***	***%***
Positive references	16	69,6%
Improvement in logistic issues (time schedule, personnel involved)	3	13,0%
Improvement in communication between Centres/personnel	3	13,0%
Improvement in communication with parents	1	4,3%
Other issues (this comment reports on difficulties with oral therapy for this child)	1	4,3%

## Discussion

Literature offers evidence of the safety of home-delivered chemotherapy for both adult and pediatric cancer and research on this topic dates back to the Nineties. In 1996, an Australian experience described 1688 visits for the administration of anticancer treatment to 179 adult patients over 5 years, reporting only rare and minor complications, and a single severe complication [14]. In 2006, a Canadian experience on 23 children with ALL who received chemotherapy at home and in the hospital did not show any difference in adverse events rate for the two groups [15]. A more recent Canadian study with 136 children (1701 visits between 2013 and 2015, 58% of which for chemotherapy administration) did not report any adverse event during home chemotherapy [9]; the same finding was that of a Danish experience with 57 children (317 chemotherapy doses administered at home) [16].

The data of our study are in line with these findings, with no adverse event occurring in the time reference interval, providing an excellent safety profile of home-delivered chemotherapy.

As for the broader topic of home assistance for pediatric oncology patients, our experience recalls that of the Genoa group, which offered supportive treatment to patients with hematological malignancies and after hematopoietic stem cell transplant but did not provide home intravenous chemotherapy [10, 11, 17]. Other teams in Italy reported on the provision of supportive treatment and end-of-life care at home settled by the Reference Oncology Team in the Hub Centre throughout cooperation with Spoke hospitals [18, 19].

As for the possibility of maintaining a family routine, literature provides evidence that a home chemotherapy program helped families maintain their routine, limiting the physical and mental burden of hospital access and helping children in maintaining a sense of normality [9, 20]. However, Stevens et al. reported that children receiving home chemotherapy tended to experience more distress, hypothesizing that in the long term children may have perceived that home was no longer a safe place, free from medical interventions [15].

Childhood cancer has a high impact on healthy siblings, both in the short and in the long term [21]. Siblings may be overlooked because of their ill brother/sister and may find it hard to cope with their parents’ difficulties [22]; moreover, some of them may experience school absenteeism, academic struggles and social restrictions from peers [23].

Our data are coherent with these literature findings. Most families reported great appreciation for the possibility of reducing the time burden for hospital access with the home chemotherapy program, and maintaining a daily work/domestic routine, with less than five hospital accesses a month. Moreover, they favorably evaluated the opportunity for siblings to experience fewer interferences in their routine and to become familiar with their brother/sister’s disease and treatment.

We adopted a simplified questionnaire with problem-oriented questions to minimize the dropout rate. We did not explicitly direct our questions to children and healthy siblings because there were many pre-school children to avoid interpretation bias.

In the comment section, some parents expressed the importance of a continual interface between the Oncology Hub Centre and the PHCT to provide consistent communication. This point is crucial for the success of such projects. Hub Centers must provide the treatment schedule with doses and rate of administration, and they should always be available to guide in the safe management of adverse effects. The PHCT should provide constant feedback on their evaluation of patients.

Home care may help the staff in developing a broader observation of patients and families in their environment: patients and families may perceive the staff as more focused and establish a closer relationship with them [24].

All families suggested expanding the program to all children diagnosed with cancer in the Region.

As far as cost analysis is concerned, literature provides favorable evidence on the financial impact of home care assistance programs [7, 11].

The daily cost of a single inpatient treatment for acute lymphoblastic leukemia is € 969,00 in the Hub Oncology Centre. On the contrary, the daily cost of a single home access for chemotherapy delivery is € 134,34 for a vincristine injection, € 136,44 for a vinblastine injection and € 138,54 for a cytarabine injection. A pediatrician and a pediatric nurse are always involved in chemotherapy administration, and they use a car purchased explicitly for the program. We speculate that the travel cost for a single home access is € 10. However, it is important to consider that the PHCT provides visits and procedures other than chemotherapy delivery to other patients in the same area, for a mean rate of six visits per day. This organization implies that it is difficult to extrapolate the exact travel cost for a single access.

Most families positively evaluated the possibility of reducing the financial burden of hospital access. We hypothesize that the project might help reducing expenses for families due to fewer travels and less personal days taken from work for both parents, but also missed daycare or school days for healthy siblings. Financial disruption could have a substantial influence on parental distress during and after treatment of childhood cancer and must be carefully considered [25, 26].

This study has some limits. We acknowledge its retrospective nature, the limited sample size, and the fact that we did not adopt a validated scale for quality of life measurement. We also recognize that asking families to recall their experience with the home chemotherapy program from as long as 9 years ago may cause a recall bias. Furthermore, we did not investigate the psychological impact on the families and the operators’ perspective.

The points of strengths are an extended time window (8 years), the inclusion of different diagnostic groups (leukemias and solid tumors) and the analysis of specific issues related to the impact of childhood cancer on family life.

Furthermore, this is the first published report regarding a home chemotherapy program for children with cancer in Italy. The National Health System in Italy ensures full coverage of direct medical expenses for patients with cancer, with foundations and charities often providing additional financial support. Nevertheless, all families must face other costs. Our findings suggest that home-delivered chemotherapy may help reduce this burden. Ultimately, this is an example of cooperation and integration between Hub Centers and peripheral hospitals, which could result in resource rationalization and cost reduction for both families and the Health System.

Our sample covered years between 2011 and 2019: during data collection and analysis, the pandemic COVID-19 hit Italy, as it did in the whole world, posing unique challenges to the health care services [27]. The enormous effort of the National Health System in Italy pointed out the need for new organization models, especially for children with cancer and other complex chronic conditions. In this unprecedented scenario, a home chemotherapy program looks extremely safe and useful, since it reduces the need for travelling and hospital stay, thus reducing the risk of infection for both children and families and limiting hospital overcrowding.

Future trends of research should address cost-effectiveness with more specific outcome measures, the effect of home-delivered chemotherapy on children and families prospectively and include a higher number of patients with solid tumors.

## Conclusions

This study shows that a home chemotherapy program is safe, feasible and useful for maintaining a good quality of life for children and families.

## Supplementary Information

**Additional file 1: Supplementary file 1.** Full questionnaire in English.

**Additional file 2: Supplementary file 2.** The blue area represents the area covered by the Pediatric Home Care Team. Padova (Veneto) and Trieste (Friuli Venezia Giulia) are the two Hub Oncology Centers. Pordenone (Friuli Venezia Giulia) is the town in which the service is based at the local hospital. Adapted from: https://www.mapsdirections.info/it/. Last accessed on June 17, 2020.

## Data Availability

Data that support the findings of this study are available on reasonable request from the corresponding author.

## Abbreviation

PHCT: Pediatric Home Care Team
ASFO: Azienda Sanitaria Friuli Occidentale
CVC: Central Venous Catheter
IQR: Central Venous Catheter
ALL: Acute Lymphoblastic Leukemia
