# Supplementary material for: Safety and family satisfaction of a home-delivered chemotherapy program for children with cancer
Source: Ital J Pediatr. 2021 Feb 26;47:43. doi: 10.1186/s13052-021-00993-x (PMC7908006; doi:10.1186/s13052-021-00993-x)
Supplement: Supplementary file 1 — Additional file 1: Supplementary file 1. Full questionnaire in English. [file 13052_2021_993_MOESM1_ESM.docx]

**Questionnaire**

Home chemotherapy for children with cancer

A retrospective analysis of safety, satisfaction and financial evaluation

Who was the parent of reference for the child’s health care?

- Mother
- Father
- Other

Family’s nationality:

- Italian
- Other

Language used with the Pediatric Home Care Team:

- Italian
- English
- Other

Profession of the reference family member:

- Housewife
- Workman
- Farmer/craftsman
- Office worker
- Executive manager
- Teacher
- Self-employed
- Entrepreneur
- Retired

Profession of the spouse:

- Housewife
- Workman
- Farmer/craftsman
- Office worker
- Executive manager
- Teacher
- Self-employed
- Entrepreneur
- Retired

Education level of the reference family member

- Primary school diploma
- Middle school diploma
- High school diploma
- Professional school license
- University degree

Education level of the spouse:

- Primary school diploma
- Middle school diploma
- High school diploma
- Professional school license
- University degree

Total number of family members (including parents)

Number of other children (excluding the child assisted by the Pediatric Home Care Team)

Did you employ a babysitter for the assistance?

- Yes
- No

If the answer is “yes,” please specify the hourly wage and the estimated number of monthly hours worked by the babysitter during the months covered by Home Care Assistance.

Please indicate the preferred mean of transport used when traveling for visits, clinical tests, and therapies to the Hub Oncology Centre and the hospital closest to your home.

- On foot
- Car
- Taxi
- Train/bus

Please indicate the average monthly frequency of travels, due to visits, clinical tests, and therapies to the Hub Oncology Centre and the hospital closest to your home.

Please indicate the distance in kilometers from your house to the nearest hospital.

Please indicate the distance in kilometers from your house to the Hub Oncology Centre.

Please indicate the average number of absences from work due to visits, clinical tests and therapies during chemotherapy at the Hub Oncology Centre and the hospital closest to your home

Has the parent of reference benefited from paid leave from work?

- Yes
- No

Has the family benefited from financial support from social services or charities during the period of therapies?

- Yes
- No

The age of the child at the time of diagnosis

Satisfaction and improvement points

Do you feel satisfied with the possibility for the parent of reference to maintain a work/home routine, (without excessive interruptions related to the therapy)?

- Very satisfied
- Satisfied
- Moderately satisfied
- Slightly satisfied
- Not at all satisfied

Do you think this type of organization has facilitated the spouse in maintaining his/her working habits? (for example, not having to ask for work permits to accompany his/her son and spouse to the hospital)

- A lot
- Enough
- A little
- Not at all

As for the use of the family car, do you think that doing chemotherapy at home has facilitated family members in its use?

- A lot
- Enough
- A little
- Not at all
- We have more than one car

Which family member was mainly present during therapy and visits?

- Mother
- Father
- Grandmother
- Grandfather
- Other

How satisfied are you overall with the possibility for the family to reduce the number of visits to the hospital by using home care assistance?

- Very satisfied
- Satisfied
- Moderately satisfied
- Slightly satisfied
- Not at all satisfied

How satisfied are you overall with the possibility for the family to reduce the time spent on travels and hospital stays?

- Very satisfied
- Satisfied
- Moderately satisfied
- Slightly satisfied
- Not at all satisfied

How satisfied are you overall with the possibility for the family to reduce the financial burden for travels and hospital stays?

- Very satisfied
- Satisfied
- Moderately satisfied
- Slightly satisfied
- Not at all satisfied

How satisfied are you overall with the possibility for your child to maintain his/her play/study routine (limiting the interruptions related to the therapy?

- Very satisfied
- Satisfied
- Moderately satisfied
- Slightly satisfied
- Not at all satisfied

How do you evaluate the possibility for your child to experience chemotherapy in a non-traumatic way because it is performed in a family context?

- Very satisfied
- Satisfied
- Moderately satisfied
- Slightly satisfied
- Not at all satisfied

How satisfied are you with the possibility for the other brothers/sisters to maintain their activities without excessive interruptions (for example, linked to accompanying their brother/sister to the hospital, so as not to remain alone at home)?

- Very satisfied
- Satisfied
- Moderately satisfied
- Slightly satisfied
- Not at all satisfied

How satisfied are you with the possibility for the other brothers/sisters to become familiar with their brother/sister’s course of therapy and to be supportive?

- Very satisfied
- Satisfied
- Moderately satisfied
- Slightly satisfied
- Not at all satisfied

How satisfied are you overall with the feeling of safety concerning the management of chemotherapy administered at home (compared to the same therapy administered in the hospital)?

- Very satisfied
- Satisfied
- Moderately satisfied
- Slightly satisfied
- Not at all satisfied

How do you evaluate overall home chemotherapy to maintain a good quality of life?

- Very useful
- Useful
- Moderately useful
- Slightly useful
- Not useful at all

Based on your experience, would you recommend extending the home chemotherapy program to all children in the region?

- Definitely yes
- Yes
- May be yes
- No
- Definitely no

This space is at your disposal for further suggestions regarding the possible improvement of the management of home chemotherapy services.
