# Supplementary figures and images for: Safety and family satisfaction of a home-delivered chemotherapy program for children with cancer
Source: Ital J Pediatr. 2021 Feb 26;47:43. doi: 10.1186/s13052-021-00993-x (PMC7908006; doi:10.1186/s13052-021-00993-x)

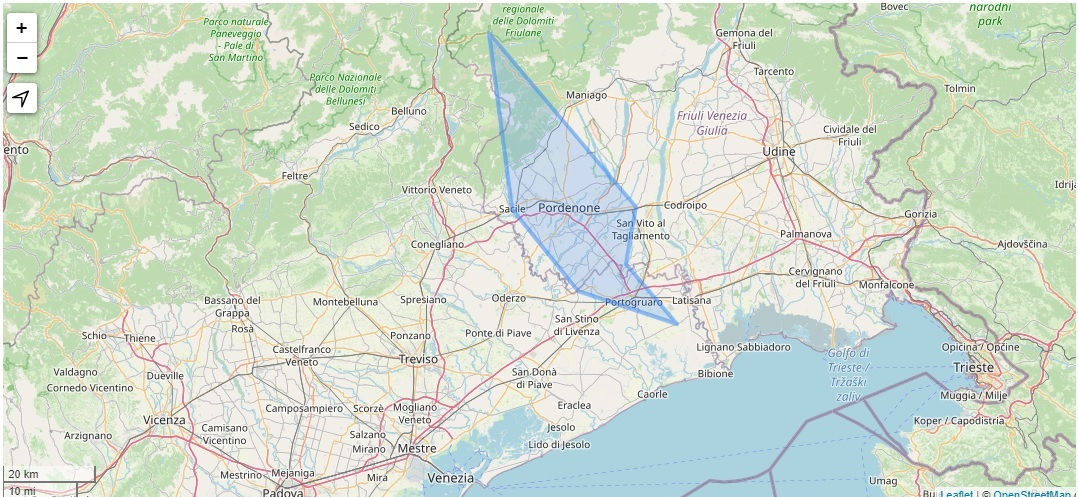

Supplement: Supplementary file 2 — Additional file 2: Supplementary file 2. The blue area represents the area covered by the Pediatric Home Care Team. Padova (Veneto) and Trieste (Friuli Venezia Giulia) are the two Hub Oncology Centers. Pordenone (Friuli Venezia Giulia) is the town in which the service is based at the local hospital. Adapted from: https://www.mapsdirections.info/it/. Last accessed on June 17, 2020. [file 13052_2021_993_MOESM2_ESM.tif]
